# Foreword

**DOI:** 10.3402/mehd.v23i0.18557

**Published:** 2012-06-18

**Authors:** Seppo Salminen

Functional Foods Forum (FFF) of University of Turku has reached an age of 10 years. The time has been full of interdisciplinary collaboration starting from food and health and terminology in the languages departments, continuing with clinical interventions studies in collaboration with the Turku University Hospital and other clinical centers, cancer research and studies on genes affecting our sensory work, and characterization of new probiotics and prebiotics as well as nutrition, flavor profiling and transfer of knowledge to SMEs. Ten years is not a long time, but through the keen collaboration and diligent work of the staff members of FFF the institute has reached an internationally recognized status.

This symposium on probiotics and prebiotics marks the 10th anniversary of the FFF. The multidisciplinary research program has involved uncovering new probiotics and prebiotics for specific uses, assessing the safety and tolerance, characterizing the properties of probiotics and prebiotics, improving the process stability of probiotics and participation in clinical intervention studies focusing on probiotic and prebiotic health effects, and most recently novel work on the nutrition economic aspects of probiotics and prebiotics. The work has resulted in collaboration over the different areas of science from languages to economics and basic microbiology and nutrition to pediatrics.

This symposium has been organized to highlight some of the areas where research has progressed during the past 10 years, to present work of some of our collaborators, and to establish a basis for future work in developing directions for new projects and collaboration.

On behalf of all of us at FFF, I would like to thank all the participants for joining forces with us for the celebration; and I hope that we shall continue to have the privilege of working with you in the future as well.

*Seppo Salminen* Professor, Director

